# Constructing In_2_S_3_/CdS/N-rGO Hybrid Nanosheets via One-Pot Pyrolysis for Boosting and Stabilizing Visible Light-Driven Hydrogen Evolution

**DOI:** 10.3390/molecules28237878

**Published:** 2023-11-30

**Authors:** Minghao Zhang, Xiaoqun Wu, Xiaoyuan Liu, Huixin Li, Ying Wang, Debao Wang

**Affiliations:** Key Lab of Inorganic Synthetic and Applied Chemistry, College of Chemistry and Molecular Engineering, Qingdao University of Science and Technology, Qingdao 266042, China

**Keywords:** In_2_S_3_/CdS/N-rGO, ultrathin nanosheets, photocatalysis, heterojunction, hydrogen evolution

## Abstract

The construction of hybrid junctions remains challenging for the rational design of visible light-driven photocatalysts. Herein, In_2_S_3_/CdS/N-rGO hybrid nanosheets were successfully prepared via a one-step pyrolysis method using deep eutectic solvents as precursors. Benefiting from the surfactant-free pyrolysis method, the obtained ultrathin hybrid nanosheets assemble into stable three-dimensional self-standing superstructures. The tremella-like structure of hybrid In_2_S_3_/N-rGO exhibits excellent photocatalytic hydrogen production performance. The hydrogen evolution rate is 10.9 mmol·g^−1^·h^−1^, which is greatly superior to CdS/N-rGO (3.7 mmol·g^−1^·h^−1^) and In_2_S_3_/N-rGO (2.6 mmol·g^−1^·h^−1^). This work provides more opportunities for the rational design and fabrication of hybrid ultrathin nanosheets for broad catalytic applications in sustainable energy and the environment.

## 1. Introduction

The photocatalytic decomposition of water to produce hydrogen has been identified as an effective and most promising strategy for dealing with environmental crises and energy scarcity [1]. Given the efficient use of solar energy, there have been many attempts in recent years to develop different photocatalysts for the decomposition of water under visible light [2]. Among which, chalcogenides and chalcogenide-based semiconductor materials have been widely investigated as photocatalysts for water splitting due to its inexpensive synthesis, low toxicity, large absorption coefficient, and narrow band gap energies [3]. The facile synthesis enables the rational design of chalcogenide catalysts with various shapes and structures and enriches the construction of hierarchical composites with other components to gain more interesting properties [4,5,6,7,8,9,10].

Cadmium sulfide (CdS) is widely used among many semiconducting metal sulfide materials due to its narrow band gap (2.4 eV), powerful reducibility, wide range of optical absorption wavelengths and a suitable energy band structure, which can effectively absorb visible light [7]. Nevertheless, CdS suffers from problems of stability in photocatalytic processes due to photocorrosion. There are still several issues with CdS that limit the rate of hydrogen production from pure CdS particles [11]. Up to now, numerous steps have been taken to address these problems [9,10,12], of which building heterojunctions with other semiconductors has been proven to be one of most the effective strategies [13,14,15]. For example, CdS/g-C_3_N_4_ nanoheterojunctions have been prepared using a hydrothermal method to improve its visible light photocatalytic performance for H_2_ production [14]. Huang et al. reported the synthesis of CdS/ZnS nanocomposites for extraordinary photocatalytic H_2_ generation via a type-II heterojunction [15]. Indium sulfide (In_2_S_3_) is an n-type semiconductor with a band gap of 2.0 to 2.4 eV, which has good stability as well as high photosensitivity [16]. Thanks to these advantages of both CdS and In_2_S_3_ semiconductors, more and more reports have focused on the synthesis of In_2_S_3_/CdS nanocomposites to form effective photocatalysts [17,18,19,20,21]. The suitable energy band structure between In_2_S_3_ and CdS benefits the construction of the In_2_S_3_/CdS heterojunction, which would accelerate the separation of photogenerated carriers and result in enhanced photocatalytic performance as compared to individual ones. For example, In_2_S_3_ nanoparticles have been deposited on CdS nanorod arrays for enhanced solar light-driven photoelectrochemical hydrogen evolution [22]. Yang et al. reported a Cu-doped In_2_S_3_/CdS heterojunction with a high spatial charge separation rate to boost photocatalytic hydrogen production [23].

However, sulfides have their inherent disadvantage of photocorrosion [24]. Thermodynamically, photocorrosion happens because of high oxidative h+ in the valence band which tends to oxidize lattice S^2−^ ions of CdS and In_2_S_3_ during the photocatalytic process. But the stability incensement could be achieved by adding a suitable sacrificial agent to scavenge the holes with competitive kinetics. For example, the photocatalytic activity and stability of sulfide photocatalysts can be efficiently improved by adding an S^2−^/SO_3_^2−^ mixture as an electron donor for hydrogen evolution from water.

On the other hand, graphene oxide has recently been introduced into semiconductor photocatalysts to further improve the efficiency of charge transfer and thereby improve photocatalytic activity and stability [25]. For example, Jia et al. reported the preparation of a N-doped graphene/CdS nanocomposite for water splitting under visible-light illumination, in which N-doped graphene could act as a protective layer to prevent photocorrosion of the CdS photocatalyst [26]. Liu et al. prepared a stacked nanostructure of GO–CdS@MoS_2_ to diminish the shortage of serious photocorrosion and obtain a high photocatalytic H_2_ evolution performance [27]. These sulfide/graphene composite preparation methods usually involve the pre-preparation of graphene and the release of poisonous substances. Thus, developing an environmentally friendly route is urgently needed to prepare sulfide/graphene composites.

Recently, deep eutectic solvents (DESs) have been accepted as novel media to apply to the fields of chemistry, materials, and catalysis because of the unique physicochemical properties including being environmentally friendly, its strong solvating ability, and its tunable compositions. More and more works have been reported exploring the potential of using DESs as reaction media for material synthesis, such as nanometals, zeolite-type materials, carbon materials, and metal–organic frameworks [28,29].

**Figure 1:**
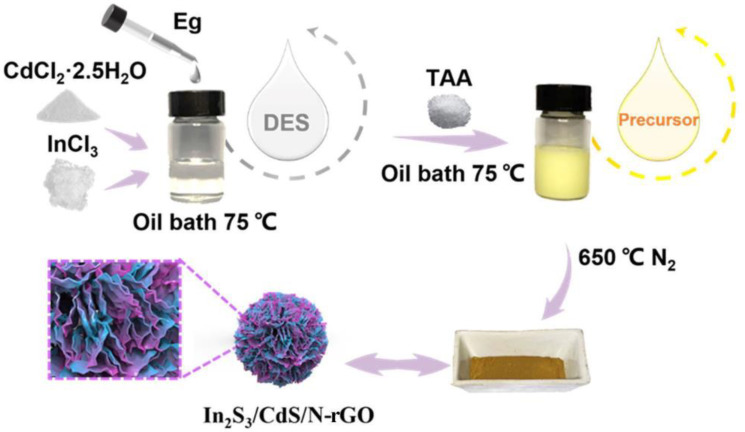
A schematic diagram for the preparation of the In2S3/CdS/N-rGO hybrid.

In this work, In and Cd-containing DES liquids were elaborately designed and applied to the synthesis of a hybrid In_2_S_3_/CdS/N-rGO photocatalyst via one-step pyrolysis, which is schematically shown in Figure 1. The unique liquid property of the DES precursor results in the in situ formation of tightly coupled interfaces in the hybrid. The in situ formed heterojunction with a spherical tremella-like structure could contribute to more active sites, highly efficient transfer and separation of photogenerated carriers, and the acquisition of strong redox stability. Under visible light, the hydrogen production rate of the In_2_S_3_/CdS/N-rGO photocatalyst achieved 10.9 mmol·g^−1^·h^−1^, which was 2.9 times that of a single component CdS and 4.2 times that of an In_2_S_3_ photocatalyst.

## 2. Results and Discussion

### 2.1. Formation of DES

**Figure 2:**
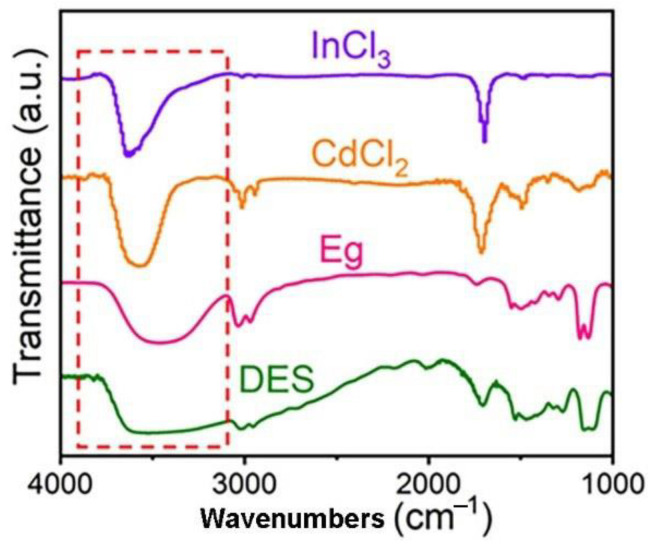
FT-IR spectra of CdCl_2_·2.5H_2_O, InCl_3_, ethylene glycol, and DES.

The formation of deep eutectic solvents (DESs) of CdCl_2_·2.5H_2_O, InCl_3_, and ethylene glycol was first characterized. As shown in Figure 1, we collected FT-IR of CdCl_2_∙2.5H_2_O, InCl_3_, ethylene glycol, and DESs. After DES formation, the chemical shift of –OH decreased because of hydrogen bond formation between Cl^−^ ions and the hydrogen atom in the DESs [30]. The absorption peak at 3429 cm^−1^ corresponds to the stretching of –OH in ethylene glycol. After DES formation with CdCl_2_·2.5H_2_O and InCl_3_, the -OH was chelated by Cl^−^. As a result, the –OH band moved to a lower wavenumber and significantly widened, which is evidence of hydrogen bond formation, enabling the components to be tightly coupled at the molecular level. At the same time, the oil bath temperature for the reaction to form a transparent and uniform liquid is about 75 °C. The melting point of the mixture was lower than the melting point of each component, which can also reveal the formation of DESs (the melting point of InCl_3_ is 586 °C, and CdCl_2_·2.5H_2_O is 568 ℃) [31]. The obtained DES liquids were used as precursors to synthesize the In_2_S_3_/CdS/N-rGO photocatalyst via a one-pot pyrolysis, as schematically shown in Figure 2.

### 2.2. Material Characterization

**Figure 3:**
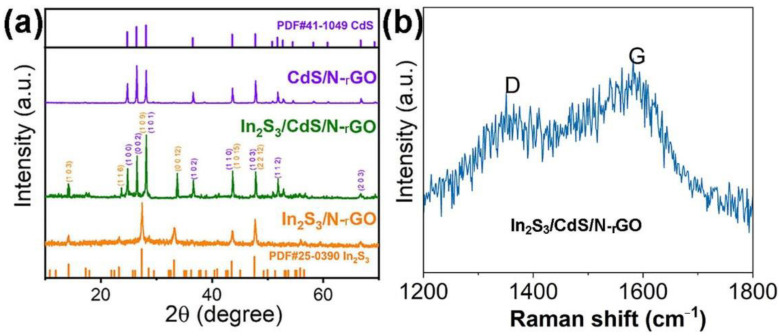
XRD patterns (**a**) and Raman spectrum (**b**) of the photocatalysts.

The structure of the as-prepared photocatalysts was identified from the respective XRD patterns. As shown in Figure 3a, the XRD of the obtained In_2_S_3_/CdS/N-rGO photocatalyst shows significant characteristic diffraction peaks at 24.81°, 26.51°, 28.18°, 36.62°, 43.68°, 47.84°, 51.82°, and 66.77° corresponding to the (100), (002), (101), (102), (110), (103), (112), and (203) crystal faces of CdS (JCPDS, No. 41–1049). The diffraction peaks at 14.25°, 23.32°, 27.43°, 33.23°, 43.60°, and 47.70° are ascribed to the (103), (116), (109), (0012), (1015), and (2212) crystal faces of In_2_S_3_ (JCPDS, No. 25–0390). The narrow and sharp characteristic peaks indicate CdS and In_2_S_3_ samples have high crystallinity. In addition, In_2_S_3_ in the hybrid has a peak shift to a higher diffraction angle. It may come from the doping of smaller N or C atoms into In_2_S_3_ or from the formation of strong interface interactions between In_2_S_3_ and CdS and even the formation of a CdIn_2_S_4_ phase [20]. The XRD results support the presence of CdS and In_2_S_3_ substances. But there are no obvious diffraction peaks of graphene in the pattern, presumably due to the relatively weak intensity in comparison with the high crystallinity of In_2_S_3_ and CdS. The Raman spectrum was recorded to further identify the presence of graphene carbon. Figure 3b shows the Raman spectrum of the catalyst with two characteristic peaks around 1350 and 1580 cm^−1^, confirming the presence of reduced graphene oxide carbon in the In_2_S_3_/CdS/N-rGO hybrid [25]. It is also evident that the Raman data are very noisy and the D band intensity is rather strong, which could be deduced from the lower content of rGO carbon in the hybrid, the doping of N atoms into rGO carbon, and the larger quantity of defects in rGO carbon because of the composition of In_2_S_3_/CdS in the in situ pyrolysis process.

**Figure 4:**
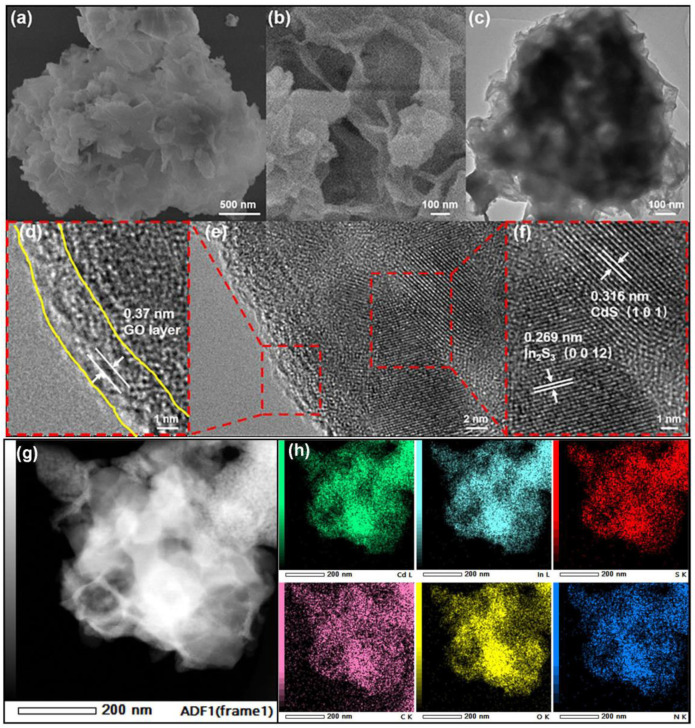
SEM (**a**,**b**), TEM (**c**), HRTEM (**d**–**f**), and HAADF-STEM image (**g**) and corresponding STEM-EDS elemental mappings (**h**) of In_2_S_3_/CdS/N-rGO photocatalyst.

The morphology and surface microstructure of the In_2_S_3_/CdS/N-rGO photocatalyst were studied using SEM and TEM. As shown in Figure 4a, the In_2_S_3_/CdS/N-rGO photocatalyst has a layered shape of tremella, with a diameter of about 1–2 µm. The tremella shows staggered growth of ultrathin curved nanosheets. These nanosheets cluster together in different directions to form open cavities distributed across the entire surface of the microspheres (Figure 4b). This open cavity will produce rich voids and a large specific surface area and expose more active sites. It can also be seen from the TEM image in Figure 4c that the contrast between light and dark indicates the porous structure of the tremella microspheres composed of ultrathin nanosheets. The microstructure of the nanosheet was further characterized by the HRTEM image in Figure 4e. After zooming in (dotted red line regions), multiple layers of an almost transparent layer at the edge of the nanosheet can be identified, as marked by yellow lines. The lattice fringe of about 0.37 nm matches the distance between the layers of graphene oxide [30]. And Figure 4f shows two types of distinct lattice fringes, the 0.269 nm lattice matching (0012) the faces of In_2_S_3_ [32] and the 0.316 nm lattice for (101) the crystal surfaces of CdS [33]. Figure 4g shows a HAADF-STEM image of the hybrid, further confirming the tremella-like structure assembled by ultrathin nanosheet. The corresponding STEM-EDS elemental mappings in Figure 4h reveal a uniform distribution of Cd, In, S, C, O, and N elements in the In_2_S_3_/CdS/N-rGO hybrid. It can be concluded that the In_2_S_3_/CdS photocatalyst coated with a graphene oxide shell can be successfully prepared by the DES precursor-assisted one-pot pyrolysis method. The N-doped graphene oxide shell can inhibit the photocorrosion of metal sulfide to a certain extent and accelerate the electron transfer of charge at the interface, which would greatly improve the photocatalytic activity and stability.

**Figure 5:**
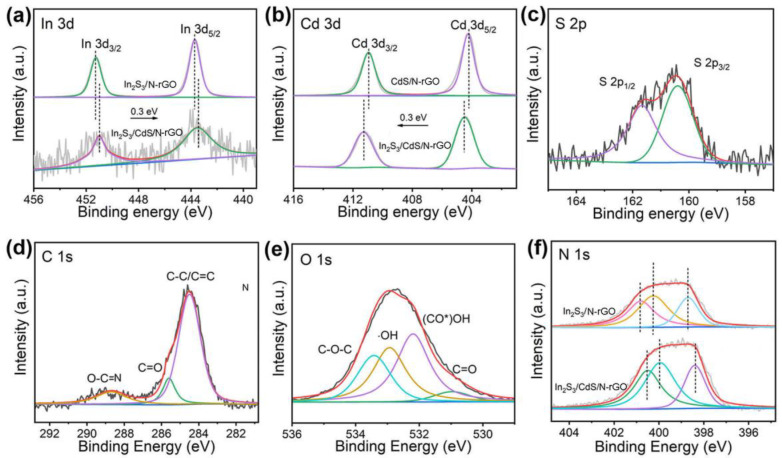
XPS spectra of (**a**) In 3d; (**b**) Cd 3d; (**c**) S 2p; (**d**) C 1s; (**e**) O 1s; and (**f**) N 1s of different photocatalysts.

Figure 5 shows XPS spectra of In 3d, Cd 3d, S 2p, C 1s, O 1s, and N 1s for different photocatalysts, respectively. The 443.4 eV and 451.0 eV peaks in Figure 5a match the In 3d_5/2_ and In 3d_3/2_ binding energy, indicating the presence of an In^3+^ state in the hybrid [34]. The characteristic peaks at 404.4 and 411.2 eV in Figure 5b correspond to the Cd 3d_5/2_ and Cd 3d_3/2_ binding energy of a Cd^2+^ valence state [35]. As shown in Figure 5c, the S 2p spectrum can be fitted into two peaks at 160.4 eV and 161.6 eV, attributed to the characteristic peaks of S 2p_3/2_ and S 2p_1/2_ of S^2−^ [36]. The C 1s spectrum of In_2_S_3_/CdS/N-rGO is shown in Figure 5d. The characteristic peak at 284.5 eV corresponds to sp^2^ hybridization carbon and carbon atoms single- or double-bonded to the nitrogen atoms or oxygen [37]. The peaks at 285.6 and 288.7 eV correspond to the C–C/C=C and O–C=N/C-N functional groups [38]. The O 1s peak can be deconvoluted into four peaks at 530.9 eV, 532.1 eV, 533 eV, and 533.4 eV (Figure 5e), which come from surface-absorbed OH groups on In_2_S_3_/CdS as well as C=O, (CO*)OH, and C–O–C groups remained in rGO [39]. Figure 5f shows the In_2_S_3_/CdS/N-rGO photocatalyst and In_2_S_3_/N-rGO photocatalyst with respect to N 1s spectra, which can further prove the existence of N elements in In_2_S_3_/GO. In_2_S_3_/CdS/N-rGO shows three weak peaks. The three peaks are located at 400.5 eV, 400.13 eV, and 398.34 eV, which correspond to graphitic nitrogen, N of pyrroline, and pyridinic N [37,40]. These nitrogen-containing functional groups can confirm the existence of N-doped reduced graphene oxide. In addition, it is worth noting that compared to the binding energy of In 3d and N 1s in the composite photocatalyst In_2_S_3_/CdS/N-rGO, the elements in the In_2_S_3_/N-rGO photocatalyst move 0.3 eV in the direction of low binding energy, while the Cd 3d in the composite moves about 0.3 eV in the opposite direction. This indicates that a close coupling interface is formed between In_2_S_3_ and CdS, attributed to the homogenous contacting of components in the liquid DES precursor.

**Figure 6:**
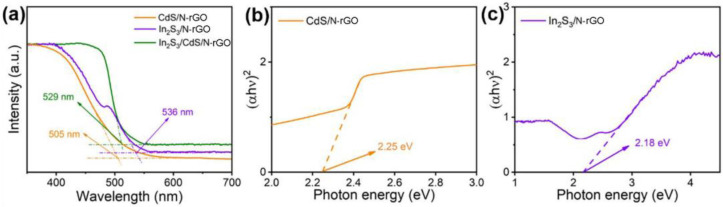
UV-Vis DRS spectra of different photocatalysts (**a**); corresponding Tauc plots for band gap estimation of (**b**) CdS/N-rGO and (**c**) In_2_S_3_/N-rGO.

In order to investigate the reason for improved photocatalytic hydrogen activity, a series of optical and electrochemical properties of the photocatalysts were measured to evaluate the charge transfer and separation ability. To investigate the optical absorption properties, UV-Vis diffuse reflectance spectroscopy (DRS) was carried out, as shown in Figure 6a. CdS/N-rGO exhibited a significant absorption edge at about 505 nm, suggesting good visible light absorption. Meanwhile, the absorption edge of the In_2_S_3_/N-rGO photocatalyst was close to 536 nm. After the In_2_S_3_/N-rGO was compounded with CdS/N-rGO, the absorption edges of In_2_S_3_/CdS/N-rGO (529 nm) fell well between those of CdS/N-rGO and In_2_S_3_/CdS/N-rGO, indicating that they can harvest more photon efficiency than CdS/N-rGO in the visible region via the compounding of In_2_S_3_/N-rGO. This result may be due to the narrow band gap and porous structure of In_2_S_3_ with a large specific surface area. It improves the utilization efficiency of sunlight and causes changes in the basic process of electron hole pair formation, improving photocatalytic performance.

The Tauc plots calculated by UV-Vis spectra through the Kubelka–Munk formula are shown in Figure 6b,c to estimate the band gaps of CdS/N-rGO and In_2_S_3_/N-rGO. The band gap energies (*Eg*) of the catalyst can be calculated by using the Tauc plot. According to previous reports, the band gap energies can be calculated by the equation (*αhν*) = *K*(*hν − Eg*)^0.5^ [41], where *α* is the absorption coefficient, *ν* is the optical frequency, *K* is a constant, and *Eg* is the band gap. By tangent to the X-axis, the band gaps of CdS/N-rGO and In_2_S_3_/N-rGO are estimated to be 2.25 eV and 2.18 eV, respectively.

### 2.3. Photocatalytic Hydrogen Evolution

**Figure 7:**
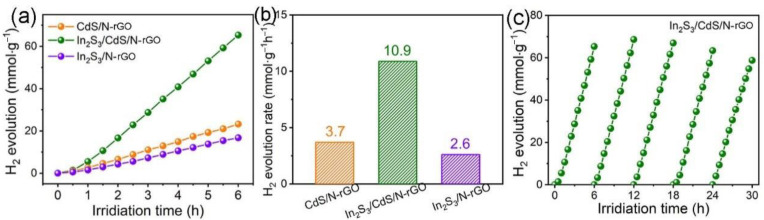
(**a**) Photocatalytic H_2_ evolution and (**b**) H_2_ evolution rates of different catalysts; (**c**) H_2_ evolution cycling stability of In_2_S_3_/CdS/N-rGO.

The photocatalytic H_2_ evolution activity of In_2_S_3_/CdS/N-rGO was investigated on an online photocatalytic system with a top light irradiation using 0.25 M Na_2_S∙9H_2_O/0.35 M Na_2_SO_3_ as sacrificial reagents. As presented in Figure 7a, the photocatalytic hydrogen evolution performance of different photocatalysts were compared. A steady accumulation of H_2_ is observed within 6 h. Figure 7b shows the corresponding hydrogen evolution rates. The In_2_S_3_/CdS/N-rGO photocatalyst has the highest value of 10.9 mmol·g^−1^·h^−1^, which is significantly improved compared to the catalytic performance of CdS/N-rGO and In_2_S_3_/N-rGO. The hydrogen evolution rate of the In_2_S_3_/CdS/N-rGO photocatalyst was 3.0 times and 4.2 times higher than that of CdS/N-rGO and In_2_S_3_/N-rGO photocatalysts, respectively. This performance is also higher than most of the non-noble metal CdS-based photocatalysts reported so far (Table 1).

In addition, the stability of photocatalysts is also one of the important criteria for evaluating the performance of catalyst materials. As shown in Figure 7c, the In_2_S_3_/CdS/N-rGO photocatalyst was used in a 30-h cycle stability experiment for photocatalytic hydrogen evolution, with a total of five cycles and each for 6 h. The results indicate that the hydrogen production performance is only slightly lower than the initial value, demonstrating that the In_2_S_3_/CdS/N-rGO photocatalyst has good cycle stability while maintaining high performance.

### 2.4. Photoelectrochemical Properties

**Figure 8:**
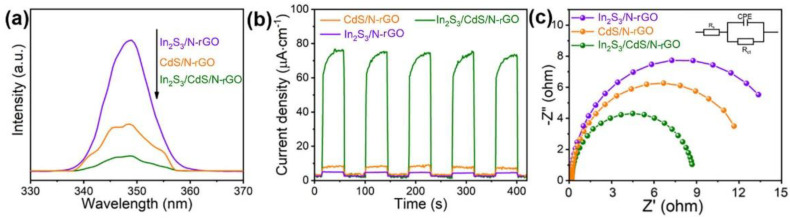
PL spectra (**a**); transient photocurrent responses curves (**b**); and EIS Nyquist plots (**c**) of different photocatalysts.

Generally, the photocatalytic performance greatly relates to the transfer and separation of photogenerated electron–hole pairs. PL intensity was applied to investigate the effectiveness of photoexcited electron–hole pair separation. As shown in Figure 8a, In_2_S_3_/CdS/N-rGO shows distinctly decreased PL intensity in comparison with CdS/N-rGO and In_2_S_3_/N-rGO. This implies that the recombination of photoinduced electron–hole pairs in In_2_S_3_/CdS/N-rGO was efficiently inhibited. In addition, transient photocurrent responses of the photocatalysts were measured by several on/off cycles under illumination (Figure 8b). After turning on the light, the I-t curve of the In_2_S_3_/CdS/N-rGO photocatalyst showed much higher photocurrent density than In_2_S_3_/N-rGO and CdS/N-rGO, indicating that the photoresponse sensitivity of photoexcited carriers could be indeed enhanced in the In_2_S_3_/CdS/N-rGO hybrid. This result is consistent with the PL results. In addition, EIS Nyquist plots are used to explore carrier dynamics, especially the charge transfer impedance at the semiconductor electrolyte interface. Figure 8c shows EIS Nyquist plots of different catalysts and the equivalent circuit model is shown in the inset. R_s_ is the solution resistance, R_ct_ represents the charge transfer resistance, and CPE is described as the constant phase element. By comparison, circle radii of In_2_S_3_/N-rGO and CdS/N-rGO are wider than that of In_2_S_3_/CdS/N-rGO, demonstrating that the internal hindrance of the In_2_S_3_/CdS/N-rGO heterojunction is less than that of the individual ingredient [35]. It is reasonable to conclude that forming an In_2_S_3_/CdS/N-rGO hybrid could greatly promote the directional migration and spatial separation of electron–hole charges from the above experiment results.

**Figure 9:**
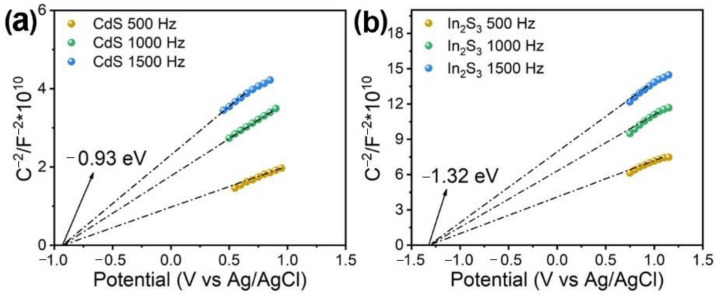
Mott–Schottky plots of (**a**) CdS/N-rGO and (**b**) In_2_S_3_/N-rGO.

Figure 9 shows Mott–Schottky plots of CdS/N-rGO and In_2_S_3_/N-rGO measured at different frequencies. Both of them show positive slope values, indicating that they are all typical n-type semiconductors. The flat band potentials (E_fb_) determined as the x-intercept in Mott–Schottky plots are −0.93 V for CdS/N-rGO and −1.32 V for In_2_S_3_/N-rGO versus Ag/AgCl. Based on the equation of E_fb_ (V vs. NHE) = E_fb_ (V vs. Ag/AgCl) + 0.61 [52], the normal hydrogen electrode (NHE) potentials were calculated to be E_fb_ (CdS) = −0.32 V and E_fb_ (In_2_S_3_/N-rGO) = −0.71 V from the flat band potentials.

### 2.5. Photocatalytic Hydrogen Evolution Mechanism

**Figure 10:**
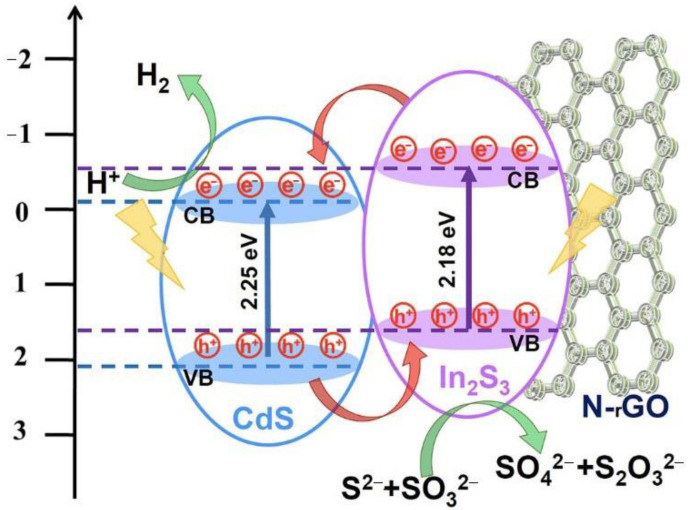
Photocatalytic hydrogen evolution mechanism of the In_2_S_3_/CdS/N-rGO photocatalyst.

It is generally believed that coupling semiconductors with different valence band (VB) and conduction band (CB) energy potentials can promote effective interfacial charge transfer. Considering that an n-type semiconductor generally has a CB bottom of about 0.2 V higher than its E_fb_ value, the CB bottom is −0.12 V for CdS and −0.51 V for In_2_S_3_ when potential difference is set as 0.2 V. And the VB tops are determined to be 2.13 V (CdS) and 1.67 V (In_2_S_3_) by adding CB potential to the band gap value obtained from Figure 6b,c. According to the above experimental results, band structures of the obtained CdS/N-rGO and In_2_S_3_/N-rGO catalysts can be displayed in Figure 10. From the band alignment, In_2_S_3_ has a higher CB edge potential than CdS, while CdS has a deeper VB maximum. As a result, a type-II heterojunction can be formed with a staggered energy band alignment at the coupled interface between CdS and In_2_S_3_ which facilitates the charge separation and transfer process [53].

According to the photochemical and photoelectrochemical experimental results discussed above, the combination of CdS and In_2_S_3_ in the type-II heterojunction could effectively accelerate the separation of photoexcited electrons and holes in space. Under visible light irradiation, photoinduced electrons in the CB of In_2_S_3_ would move to the CB of CdS, which would be captured by H^+^ ions to produce H_2_, whereas the holes transfer from the VB of CdS to the VB of In_2_S_3_, which would be captured by the sacrificial reagent. As a result, the accumulation of holes on the VB of CdS was prevented, inhibiting the photocorrosion of CdS and improving the stability of the photocatalyst. The elaborately designed DES liquid procedure enables the formation of the In_2_S_3_/CdS/N-rGO hybrid in one step, resulting in tightly coupled interfaces and an enhanced charge conductivity. In addition, the coupling of N-rGO plays significant roles in improving the photocatalytic performance. N-doping introduces electron-rich N into the graphene framework and enhances the electrical conductivity of rGO. N-doping forms additional defects and leads to a structural change in graphene carbon, which serve as active sites for the in situ growth of In_2_S_3_ and CdS, forming tightly coupled interfaces and enhancing the charge transfer of the hybrids [54]. Also, N-rGO can protect In_2_S_3_ and CdS from corrosion. Furthermore, the tremella-like structure provides more active sites for reactant species, such as hole scavengers, H^+^, and H_2_O, promoting reaction kinetics for enhanced photocatalytic activity [55]. In summary, the CdS/In_2_S_3_/N-rGO hybrid possesses a synergetic effect of visible light absorption enhancement, type-II heterojunction formation, and spherical tremella-like structure, which act together to achieve high photocatalytic performance.

## 3. Materials and Methods

### 3.1. Chemicals

Indium chloride (InCl_3_, 98%) was purchased from Shanghai Macklin Biochemical Co., Ltd. Cadmium chloride (CdCl_2_·2.5H_2_O), ethylene glycol, thioacetamide, sodium sulfide (Na_2_S·9H_2_O), anhydrous sodium sulfite, and absolute ethanol were purchased from Sinopharm Chemical Reagent Co., Ltd. All chemicals were used without further purification. Deionized water was used in all experiments.

### 3.2. Preparation of the Photocatalysts

The In_2_S_3_/CdS/N-rGO composite photocatalyst was prepared via the one-step pyrolysis method using DESs as precursors, as schematically shown in Figure 2. A total of 2.5 mmol of InCl_3_, 2.5 mmol of CdCl_2_∙2.5H_2_O, and 17 mmol of ethylene glycol were firstly mixed into a glass bottle and heated in a 75 °C oil bath. The mixture converted to a transparent and homogeneous liquid after 30 min stirring. Then, 7.5 mmol of thiourea was added, and a uniform yellow liquid was formed after stirring for another 30 min. Finally, the liquid precursor was transferred to a covered porcelain boat. Then, the porcelain boat was placed in the center of the tube furnace and heated to 650 °C at a rate of 5 °C/min under N_2_ atmosphere and kept for 4 h. After cooling to room temperature, the sample was washed, centrifuged three times with deionized water and ethanol, and dried to obtain the In_2_S_3_/CdS/N-rGO composite photocatalyst. Figure 1 schematically shows the synthesis procedure of the In_2_S_3_/CdS/N-rGO photocatalyst. CdS/N-rGO and In_2_S_3_/N-rGO photocatalysts were prepared via the same procedure, without the addition of InCl_3_ for CdS/N-rGO and in the absence of CdCl_2_∙2.5H_2_O for In_2_S_3_/N-rGO.

### 3.3. Materials Characterization

FT-IR spectra were recorded on Bruker Tensor 27 IR spectrometer and the sample was prepared by the KBr pellet method. Powder X-ray diffraction (XRD) characterizations were conducted on a D-MAX 2500/PC powder X-ray diffractometer. Scanning electron microscope (SEM) images were taken on a JSM-6700F microscope. TEM and high-resolution TEM (HRTEM) images were recorded on a JEM-F200 transmission electron microscope. XPS was conducted on a Thermo ESCALAB 250XI spectrometer. PL spectra were obtained by a Perkin Elmer LS-55. UV-Vis diffuse reflectance spectra (DRS) were conducted on a Lambda 750 s UV/VIS/NIR spectrophotometer.

### 3.4. Photocatalytic Reaction

The photocatalytic hydrogen production activity of the catalysts was evaluated on a CEL-SPH2N online photocatalytic hydrogen production system. A 300 W xenon lamp (Beijing Zhongjiao Jinyuan) equipped with 420 nm cut-off filter was applied to irradiation. By using a magnetic stirrer, 5 mg of the catalyst was dispersed in 100 mL of deionized water containing 0.25 M Na_2_S∙9H_2_O/0.35 M Na_2_SO_3_ in a quartz reactor. The system was evacuated for 25 min to remove dissolved O_2_ and CO_2_ and the temperature was maintained at 7 °C. The hydrogen production was analyzed periodically using online gas chromatography (Agilent 7890 A) with intervals of 30 min. High purity nitrogen was used as a carrier gas.

### 3.5. Photoelectrochemical Properties

Electrochemical properties were carried out on a Moudulab XM electrochemical workstation in a 0.5 M Na_2_SO_4_ solution using a standard three-electrode system, the catalyst as working electrode, an Ag/AgCl electrode as a reference, and a Pt plate as a counter electrode. To prepare a working electrode, 12 mg of the catalyst, 50 µL Nafion, and 8 mg of carbon black were dispersed in 1 mL of ethanol, ultrasonically forming a homogeneous slurry. A total of 20 µL of slurry was dropped on a slide of FTO glass with an effective area of 1 × 1 cm^2^. After being dried at 40 °C for 24 h, a working electrode was obtained. The transient photocurrent response (I-t) was performed without bias, illuminated by a 300 W xenon lamp (λ > 420 nm) switching on and off every 40 s.

## 4. Conclusions

In conclusion, a In_2_S_3_/CdS/N-rGO hybrid photocatalyst with a tremella-like structure was successfully prepared using a one-step pyrolysis method with DES liquids as precursors. The liquid DES precursor strategy has several advantages. (1) The homogeneous system enables sufficient contact between components. (2) The in situ growing and coupling of CdS/In_2_S_3_ and N-rGO were gained in one step, resulting in tightly coupled interfaces. (3) The tightly coupled N-rGO can effectively promote the rapid charge transfer and reduced electron hole recombination and can protect In_2_S_3_ and CdS from corrosion. (4) The tremella-like structure can provide more active sites. As a result, excellent photocatalytic hydrogen production performance was obtained. It can gain a high photocatalytic hydrogen production rate of 10.9 mmol·g^−1^·h^−1^. The liquid DES precursor strategy can be applied to prepare other transition metal sulfides/rGO hybrids, providing new candidates for highly efficient photocatalysts.

## Figures and Tables

**Table 1:** Comparison of the hydrogen evolution performance of the In_2_S_3_/CdS/N-rGO photocatalyst with other reported CdS-based photocatalysts.

Catalyst	Light Source	Scavengers	H_2_ Evolution Rate (mmol·g^−1^·h^−1^)	Ref.
In_2_S_3_/CdS/N-GO	λ ≥ 420 nm	Na_2_S and Na_2_SO_3_	10.9	This work
CdS-Cu_1.81_S	λ ≥ 420 nm	Na_2_S and Na_2_SO_3_	2.714	[42]
CoS/CdS	λ ≥ 420 nm	Na_2_S and Na_2_SO_3_	0.143	[43]
CdS@Zn-C	λ ≥ 420 nm	Na_2_S and Na_2_SO_3_	6.6	[44]
PbTiO_3_/CdS	λ ≥ 420 nm	Na_2_S and Na_2_SO_3_	0.849	[45]
Cd/CdS	λ ≥ 420 nm	Na_2_S and Na_2_SO_3_	1.753	[46]
Ti_3_C_2_@CdS	λ ≥ 420 nm	methanol	0.088	[47]
CdS/NiO	λ ≥ 420 nm	Na_2_S and Na_2_SO_3_	1.77	[48]
CdS/np-rGO	λ ≥ 420 nm	Na_2_S and Na_2_SO_3_	2.171	[49]
Co@NC/CdS	λ ≥ 420 nm	lactic acid	8.2	[50]
C/CdS	λ ≥ 420 nm	Na_2_S and triethanolamine	5.71	[51]

## Data Availability

The data presented in this study are available on request from the author.
